# Deep learning for automated instance segmentation of residual roots in panoramic radiographs using mask R-CNN: A retrospective diagnostic accuracy study

**DOI:** 10.1097/MD.0000000000049876

**Published:** 2026-07-24

**Authors:** Wenhui Li, Yaqin Chang, Rui Li, Min Lu, Jiao Zhang

**Affiliations:** a Department of Periodontics, Suzhou Stomatological Hospital, Suzhou, Jiangsu, China; b Department of Prosthodontics, Jiangyin Stomatological Hospital, Jiangyin, Jiangsu, China.

**Keywords:** artificial intelligence, deep learning, mask R-CNN, panoramic radiographs, residual roots, segmentation

## Abstract

**Background:**

This study aimed to evaluate the efficacy of Mask regions with convolutional neural network (R-CNN) for the automated detection and segmentation of residual dental roots in panoramic radiographs and to compare its diagnostic performance against the semantic segmentation benchmark, U-shaped network (U-Net).

**Methods:**

A retrospective dataset comprising 224 patients with 505 annotated residual roots was utilized. Image preprocessing involved adaptive contrast enhancement using contrast limited adaptive histogram equalization in the Commission Internationale de l’Éclairage lab color space to improve root-to-bone definition. A Mask R-CNN model utilizing a ResNet-50 backbone and Feature Pyramid Network was trained using K-fold cross-validation. Performance was compared to U-Net based on sensitivity, specificity, accuracy, dice similarity coefficient, and receiver operating characteristic analysis.

**Result:**

The Mask R-CNN model significantly outperformed U-Net across all evaluated metrics. It achieved an accuracy of 98.67% and a dice similarity coefficient of 91.34%. Most notably, the model demonstrated a sensitivity of 91.16%, presenting a marked improvement over U-Net (78.54%), while maintaining a specificity of 99.12%. The area under the curve was calculated at 0.9599, indicating superior discriminative capability.

**Conclusion:**

Mask R-CNN provides a robust solution for identifying residual roots, effectively addressing challenges related to low contrast and anatomical noise. By combining high sensitivity with high specificity, the system significantly reduces false negatives without causing alert fatigue, thereby serving as a reliable automated assistant for enhancing surgical safety and planning efficiency.

## 1. Introduction

The retention of residual dental roots constitutes a significant complication arising from difficult extractions, trauma, or advanced caries. While asymptomatic fragments may occasionally be submerged to preserve bone, the majority necessitate surgical removal to prevent infection and mechanical interference with prosthetics.^[1–4]^ However, detection is often complicated by trabecular bone patterns that mimic root density, particularly when the periodontal ligament (PDL) space is obscured. Consequently, precise preoperative assessment of root morphology and and proximity to vital structures is paramount for ensuring surgical safety.^[5,6]^

Historically, computer vision in dentistry relied on rule-based algorithms and low-level image processing techniques, such as active contour models and level sets, to identify dental structures. While these methods achieved moderate success in segmenting high-contrast structures like enamel-capped crowns, they frequently failed when applied to residual roots.^[7]^ Residual roots lack the distinct radiopaque enamel boundary that traditional edge-detection algorithms rely upon, and the intensity inhomogeneity of the jawbone renders these conventional “shallow learning” approaches insufficient for reliable clinical use.^[8]^ The inability of these systems to robustly handle the topological variability of fractured roots and the noise inherent in X-ray imagery paved the way for the adoption of data-driven deep learning methodologies, specifically convolutional neural network (CNN), which learn hierarchical feature representations directly from large datasets.^[9–12]^

The advent of instance segmentation architectures, particularly Mask regions with CNN (R-CNN), has revolutionized medical image analysis by combining object detection with pixel-level segmentation. Unlike semantic segmentation models such as U-shaped network (U-Net), which classify pixels into categories but cannot distinguish between individual instances of the same class, Mask R-CNN excels at identifying distinct tooth fragments as separate objects.^[13–15]^ This capability is achieved through its region proposal network (RPN) and the innovative region of interest align (RoIAlign) layer, which preserves exact spatial correspondence by eliminating the quantization errors found in previous architectures.^[16]^ By generating high-quality segmentation masks for each detected region of interest, Mask R-CNN allows for the precise localization of multiple adjacent root fragments, a critical requirement for surgical planning where the differentiation between a single fractured root and multiple comminuted fragments dictates the surgical approach.^[17–19]^

This paper investigates the application of Mask R-CNN for segmenting residual roots in panoramic radiographs (PRs), demonstrating its clinical superiority over semantic segmentation and single-stage detectors. We analyze the architectural advantages (specifically the multitask loss function) that optimize this task, while addressing critical challenges regarding data scarcity and class imbalance. Ultimately, this work underscores the shift toward automated surgical guidance systems that enhance both patient safety and operational efficiency.

## 2. Materials and methods

### 2.1. Dataset and study population

The dataset for this retrospective study consisted of dental PRs retrospectively collected from our hospital from April 2023 to October 2025. A total of 224 patients were included, comprising 100 females and 114 males, with ages ranging from 10 to 58 years. Within these images, a total of 505 residual roots were identified and meticulously annotated. Images were acquired using 2 standard panoramic units (Sirona Orthophos XG and Planmeca ProMax 2‑dimensional [2D]) operating at 71 kV, 8 mA, 14.2 seconds. The study was conducted in accordance with the Declaration of Helsinki and approved by the Ethics Committee of our hospital.

**Figure 1.**
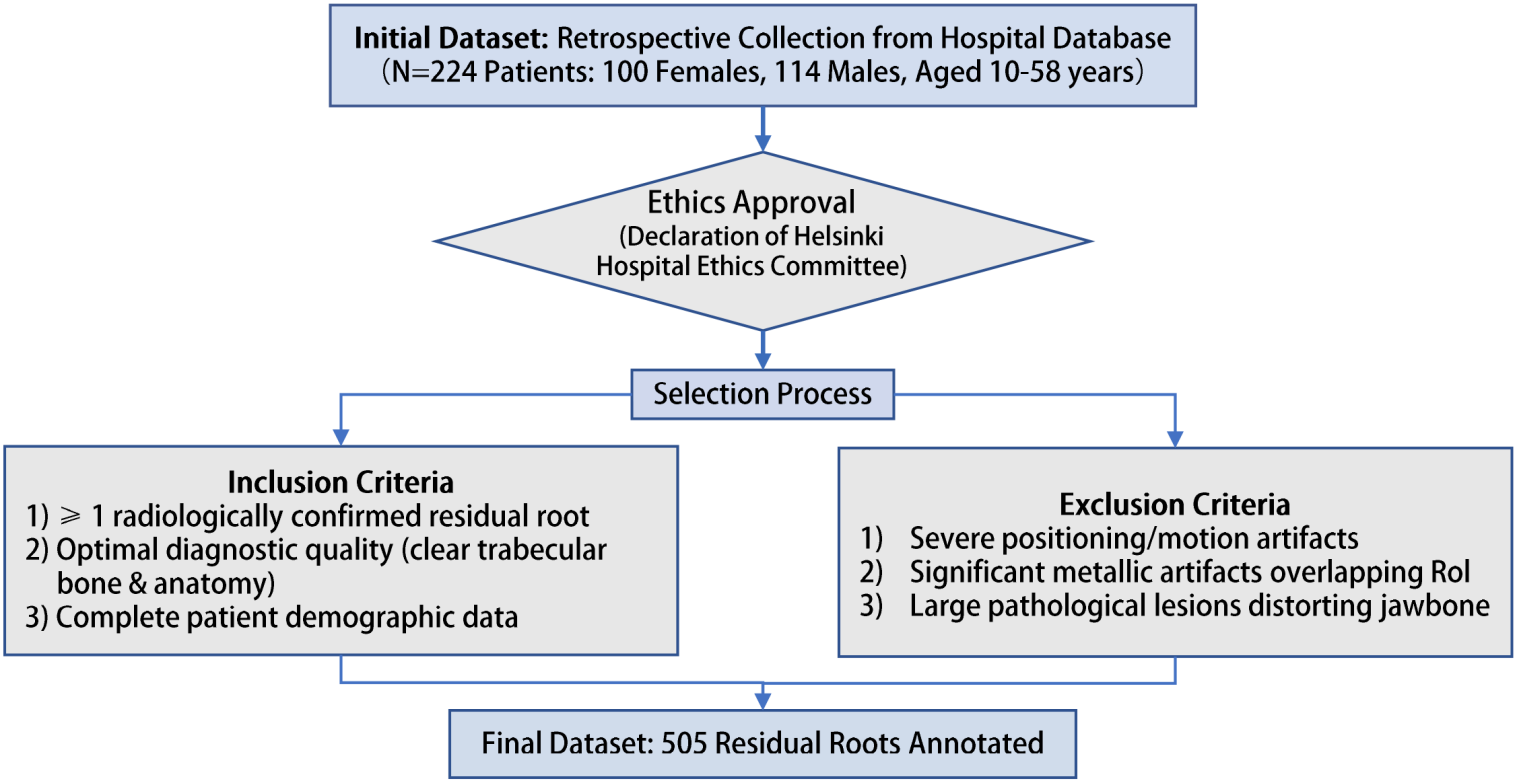
Flowchart of data selection process. N = number of patients.

The selection of radiographs followed strict clinical and technical protocols. The inclusion criteria were defined as follows: radiographs containing at least 1 radiologically confirmed residual root fragment; images with optimal diagnostic quality, ensuring clear visibility of the trabecular bone pattern and adjacent anatomical structures; and availability of complete patient demographic data. Conversely, images were excluded if they exhibited: severe positioning errors or motion artifacts (e.g., ghost images) that compromised image sharpness; the presence of significant metallic artifacts (e.g., from dental implants or extensive amalgam restorations) specifically overlapping with the region of interest; or co-existing large pathological lesions (e.g., cysts or tumors) that severely distorted the jawbone morphology (Fig. 1).

### 2.2. Ground truth annotation and range of interest (ROI) definition

**Figure 2.**
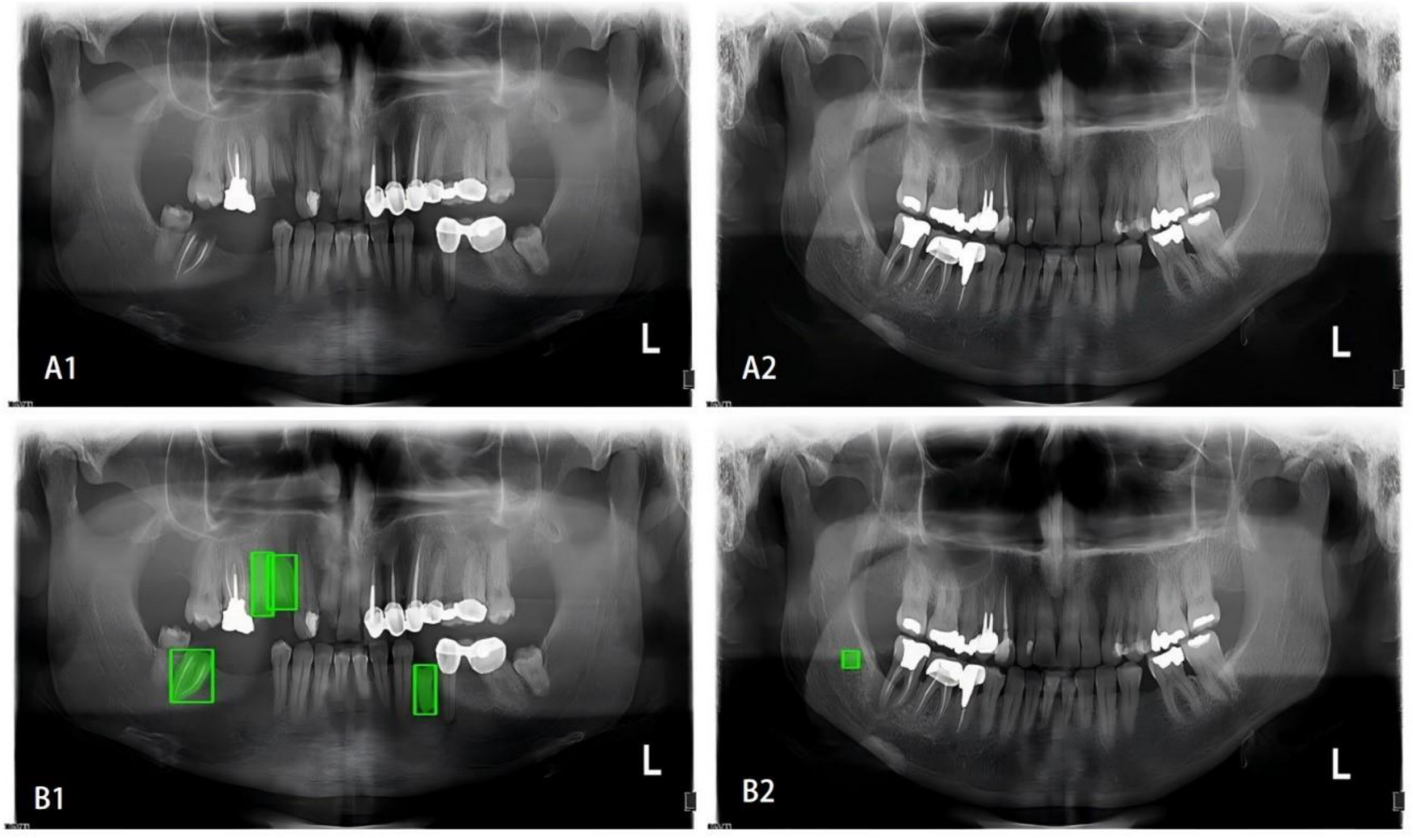
ROI selection of the study. ROI = range of interest.

To establish a precise ground truth for supervised learning, a rigorous manual annotation process was implemented. The ROIs encompassing the residual roots were delineated by 2 independent oral and maxillofacial radiologists, each with over 5 years of clinical experience. The annotation was performed using the open-source image annotation software LabelMe. Unlike standard object detection tasks that rely on bounding boxes, the Mask R-CNN architecture requires pixel-level accuracy for instance segmentation. Consequently, the experts utilized the polygon tool to meticulously trace the exact anatomical boundaries of each residual root. Special care was taken to exclude the PDL space and adjacent trabecular bone, ensuring that the ROIs represented only the root structure (Fig. 2). To ensure the reliability of the ground truth (“gold standard”), a 2-stage verification protocol was adopted. Any discrepancies regarding the existence or boundary of a root fragment between the 2 primary annotators were reviewed by a third senior professor to reach a consensus. The final polygonal coordinates were subsequently converted into binary masks and corresponding bounding boxes to serve as training targets.

To quantitatively evaluate the inter-rater reliability prior to the consensus arbitration, 2 statistical metrics were employed. Cohen kappa was calculated to assess the agreement between the 2 radiologists regarding the presence or absence of residual roots (detection agreement). Concurrently, the dice similarity coefficient (DSC) was utilized to measure the spatial overlap of their independent polygon annotations (segmentation agreement).

### 2.3. Image preprocessing and contrast enhancement

To mitigate the impact of varying X-ray exposure and anatomical density on detection accuracy, a specialized preprocessing pipeline was developed.

#### 2.3.1. Adaptive contrast enhancement via contrast limited adaptive histogram equalization (CLAHE)

The image was transformed back to the 3-channel red, green, and blue (RGB) format to meet the input layer requirements of the pretrained ResNet-50 backbone, which expects standard 3-channel image tensors. Standard RGB radiographs were first converted into the Commission Internationale de l’Éclairage Lab color space. This separation allows for the isolation of the L channel (Luminance) from the a and b channels (chromatic components). CLAHE was then applied exclusively to the L channel with a clipLimit of 2.0 and a tileGridSize of (8, 8).^[20]^ This specific configuration was chosen to enhance the local contrast at the root-to-bone interface, making the edges of residual roots more distinct while preventing the overamplification of background noise common in dental X-rays. The enhanced L channel was subsequently remerged with the original chromaticity channels and transformed back to the RGB color space.

#### 2.3.2. Data augmentation and normalization

To improve the model’s generalization across different clinical scenarios: geometric augmentation: random horizontal flipping was applied with a 50% probability to the images and their corresponding masks/bounding boxes, accounting for the bilateral symmetry of dental structures; standardization: all images were resized to a uniform resolution of 512 × 512 pixels; tensor transformation: pixel intensities were normalized from [0, 255] to a floating-point range of [0.0, 1.0]. The final data was converted into PyTorch Tensors (C, H, W) for efficient graphic processing unit.

### 2.4. Model architecture: mask R-CNN

**Figure 3.**
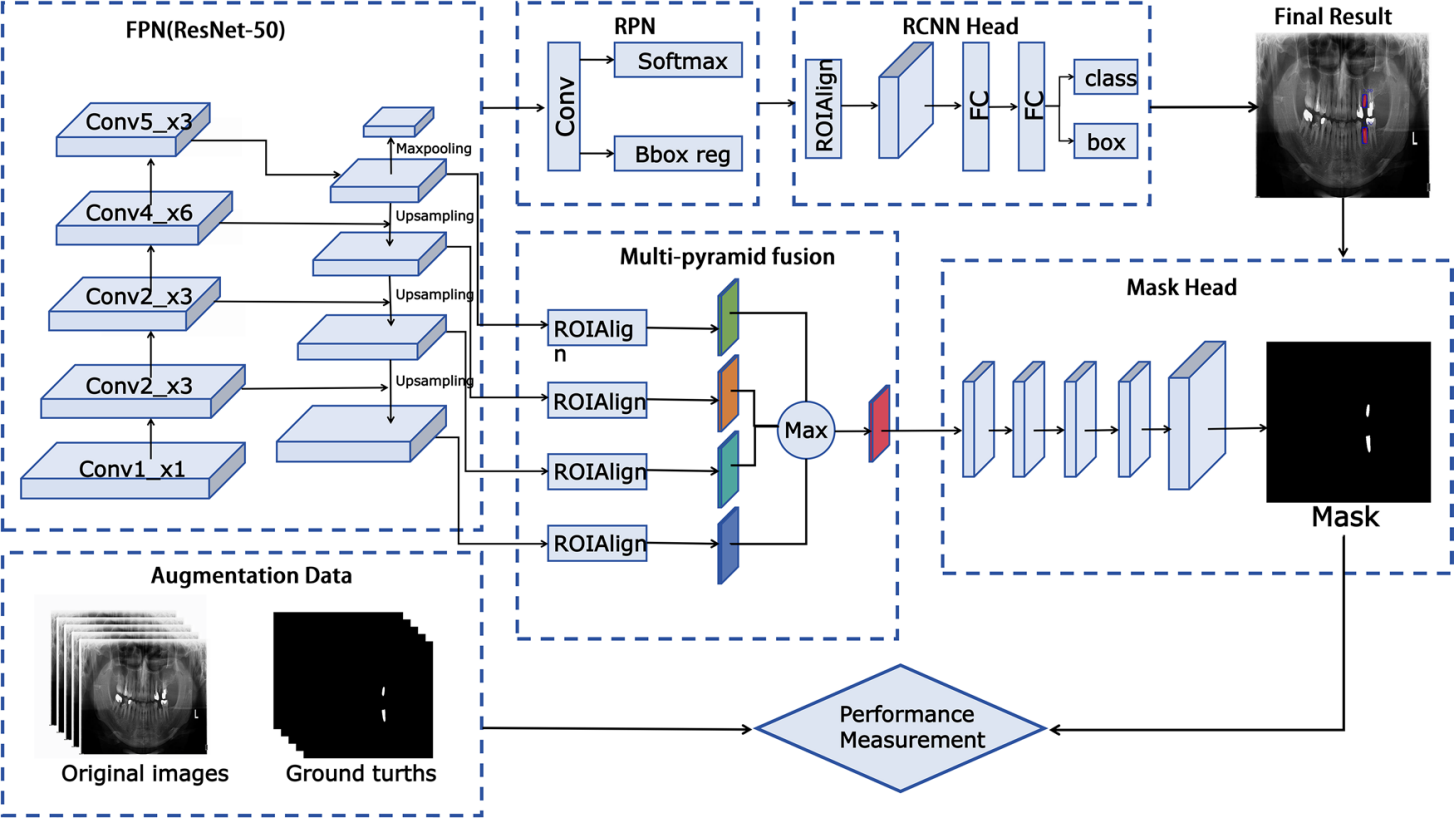
Network structure of Mask R-CNN. FPN = feature pyramid network, R-CNN = regions with convolutional neural network, RPN = region proposal network.

In this research, Mask R-CNN was employed as the core framework due to its superior performance in simultaneous object detection and instance segmentation. The architecture consists of 2 primary stages (Fig. 3)^[13,21]^:

#### 2.4.1. Feature extraction

A ResNet-50 backbone combined with a feature pyramid network is utilized to extract multi-scale feature maps. This allows the network to detect residual roots of varying sizes, from small fragments to larger structures.

#### 2.4.2. Task heads

Based on the features extracted via RoIAlign, the model branches into 3 specific heads: a classification head to identify the presence of a residual root, a regression head for bounding box localization, and a mask head for pixel-level segmentation.

### 2.5. Loss function and training

The model was trained by minimizing a multitask loss function (*L*), which integrates the classification loss (*L*_*cls*_), bounding box loss (*L*_*box*_), and segmentation mask loss (*L*_*mask*_):


$$\begin{array}{c} L=L_{cls}+L_{box}+L_{mask} \end{array}$$

Specifically, *L*_*mask*_ is defined as the average binary cross-entropy loss, ensuring that the predicted mask accurately aligns with the ground truth annotation of the residual root:


$$\begin{array}{c} L_{mask}=-\frac{1}{m^{2}}\sum_{i,j=1}^{m}\left[y_{ij}\log{\hat{y}}_{ij}^{k}+\left(1-y_{ij}\right)\log\left(1-{\hat{y}}_{ij}^{k}\right)\right] \end{array}$$

Where $y_{ij}$ represents the ground truth label and ${\hat{y}}_{ij}^{k}$ is the predicted probability for pixel (i, j) in the k-th class.

The models were trained on an NVIDIA RTX 3080Ti graphic processing unit. We utilized the AdamW optimizer with an initial learning rate of 0.001, coupled with a step decay schedule (gamma = 0.1 every 40 epochs). The batch size was set to 16. The backbone was initialized with pretrained ImageNet weights. For the RPN, we sampled 128 instances per image, and the intersection over union threshold was set to 0.5 for both region proposals and final mask evaluation.

A 5-fold cross-validation strategy was implemented to ensure the statistical reliability of the results and to maximize the use of available data for training. The entire dataset of 224 patients was randomly partitioned into 5 mutually exclusive subsets (folds) of approximately equal size. In each of the 5 folds, the dataset of 224 patients was strictly split into 179 patients (approximately 404 roots) for training and 45 patients (approximately 101 roots) for validation. This process was repeated 5 times, ensuring that every image served as validation data exactly once. Crucially, the data splitting was performed at the patient level rather than the image level. This protocol ensured that all radiographs belonging to a single patient were allocated exclusively to either the training or the validation set, thereby preventing data leakage and ensuring that the model’s generalization ability was tested on unseen anatomical structures. The final performance metrics reported in this study represent the highest values in all 5 folds.

### 2.6. Comparative analysis (U-Net)

To further validate the efficacy of our approach, the performance of the Mask R-CNN model was compared against U-Net, a benchmark architecture in medical image segmentation. U-Net utilizes a symmetric encoder-decoder structure with skip connections to preserve spatial information, which is particularly effective for identifying fine-grained lesion boundaries.^[22]^ To ensure a fair baseline, the U-Net model was trained using the exact same preprocessing pipeline, data augmentation, cross-validation folds, and hardware as Mask R-CNN.

### 2.7. Evaluation metrics

The detection and segmentation performance were quantified using 5 standard metrics: sensitivity (recall), specificity, accuracy, the DSC, and the Jaccard index (intersection over union). These metrics are calculated as follows:


$$\begin{array}{c} Sensitivity=\frac{TP}{TP+FN} \end{array}$$

$$\begin{array}{c} Specificity=\frac{TN}{TN+FP} \end{array}$$

$$\begin{array}{c} Dice=\frac{2\times TP}{FP+2\times TP+FN} \end{array}$$

$$\begin{array}{c} Jaccard=\frac{TP}{TP+FP+FN} \end{array}$$

Where TP, TN, FP, and FN represent true positives (TP), true negatives, false positives (FP), and false negatives (FN), respectively.

The diagnostic capability was also validated through receiver operating characteristic (ROC) analysis, and the area under the curve (AUC) was calculated. The ROC curve and AUC were computed on a per-instance basis. For Mask R-CNN, the continuous confidence score generated by the classification head for each predicted instance was utilized as the threshold variable against the ground truth.

## 3. Result

**Figure 4.**
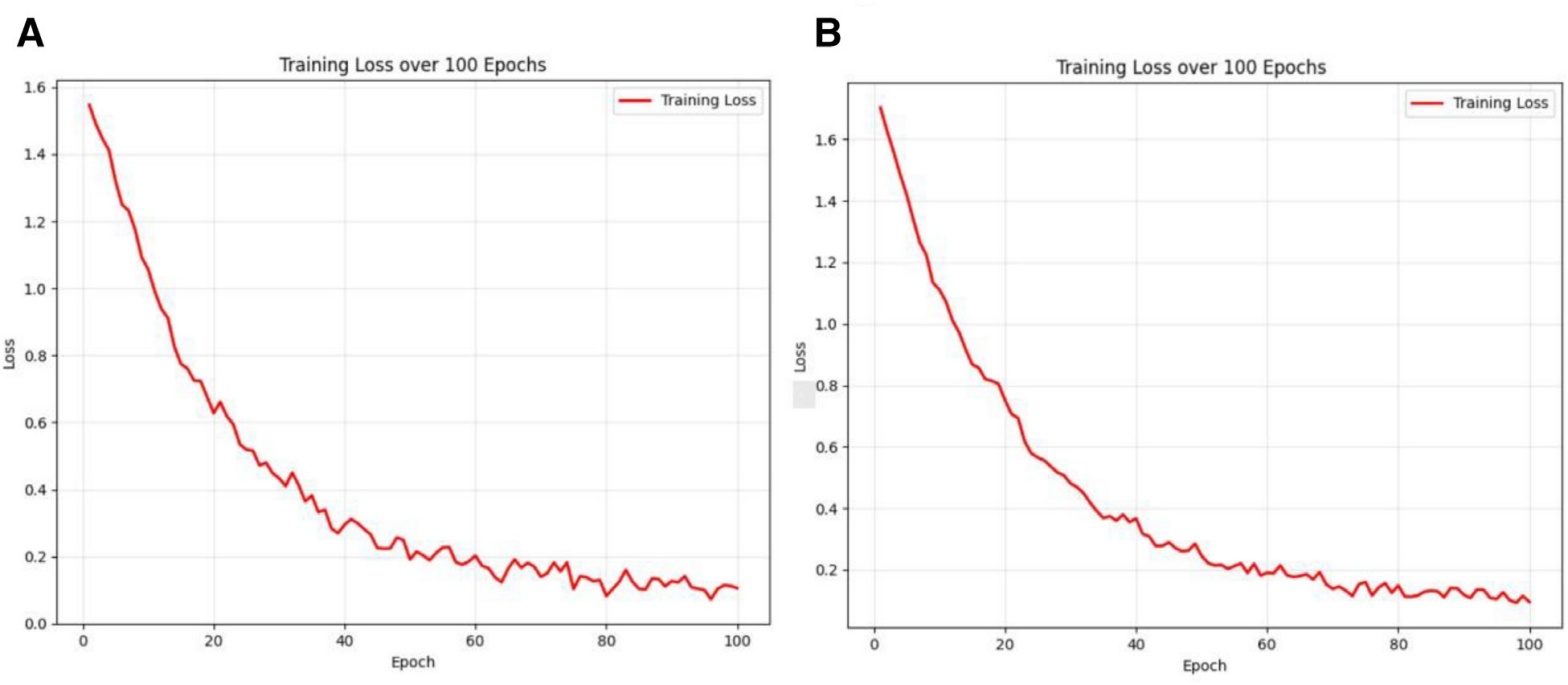
Loss curve for both models over 100 epochs. (A) Mask R-CNN, (B) U-Net. R-CNN = regions with convolutional neural network, U-Net = U-shaped network.

**Figure 5.**
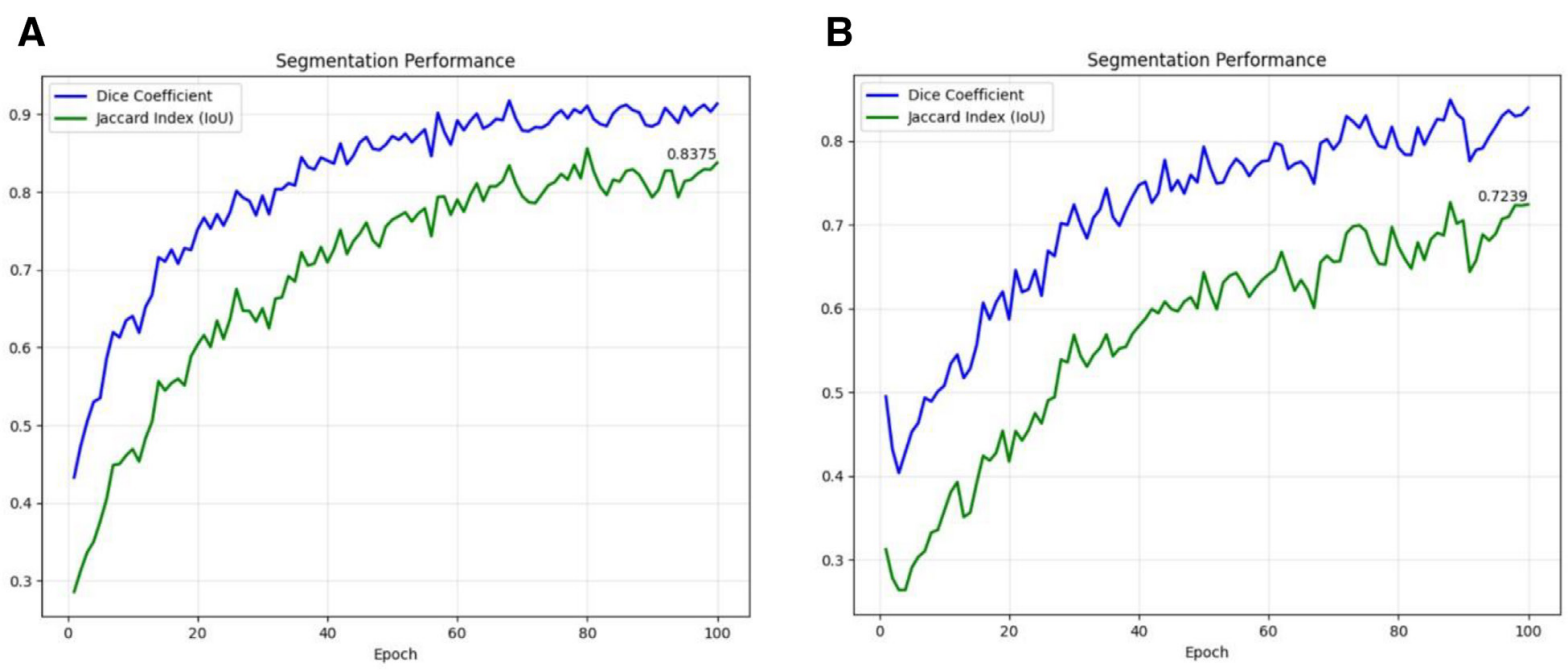
Dice coefficient and Jaccard index curves for both models over 100 epochs. (A) Mask R-CNN, (B) U-Net. IoU = intersection over union, R-CNN = regions with convolutional neural network, U-Net = U-shaped network.

The performance of the proposed enhanced Mask R-CNN model and the benchmark U-Net model was evaluated using a K-fold cross-validation approach. Table 1 summarizes the final performance metrics after 100 epochs of training. The experimental results demonstrate that the enhanced Mask R-CNN significantly outperforms U-Net across all evaluated parameters. Specifically, the proposed model achieved an Accuracy of 98.67% and a DSC of 91.34%, which are 1.87% and 7.41% higher than those of U-Net, respectively (Figs. 4 and 5).

**Table 1:** Comparative performance of Mask R-CNN and U-Net in residual root detection.

Model	Sensitivity (%)	Specificity (%)	Accuracy (%)	Dice (%)	Jaccard (%)	AUC
U-Net	78.54	96.60	96.80	83.93	72.39	0.9166
Mask R-CNN (Ours)	91.16	99.12	98.67	91.34	83.75	0.9599

AUC = area under the curve, R-CNN = regions with convolutional neural network, U-Net = U-shaped network.

### 3.1. Inter-rater reliability and annotation consensus

Prior to the third-party arbitration, the 2 independent radiologists demonstrated nearly perfect diagnostic and anatomical agreement. The inter-rater reliability for root detection yielded a Cohen kappa of 0.98, while the spatial overlap of the initial annotations achieved a mean Dice coefficient of 0.91. The details of the inter-rater agreement are summarized in Table 2.

**Table 2:** Inter-rater reliability metrics for residual root annotation prior to arbitration.

Parameter	Metric/Value
Detection Agreement	Cohen Kappa = 0.98
Segmentation Agreement	Mean Dice Coefficient = 0.91

### 3.2. Detection and segmentation accuracy

**Figure 6.**
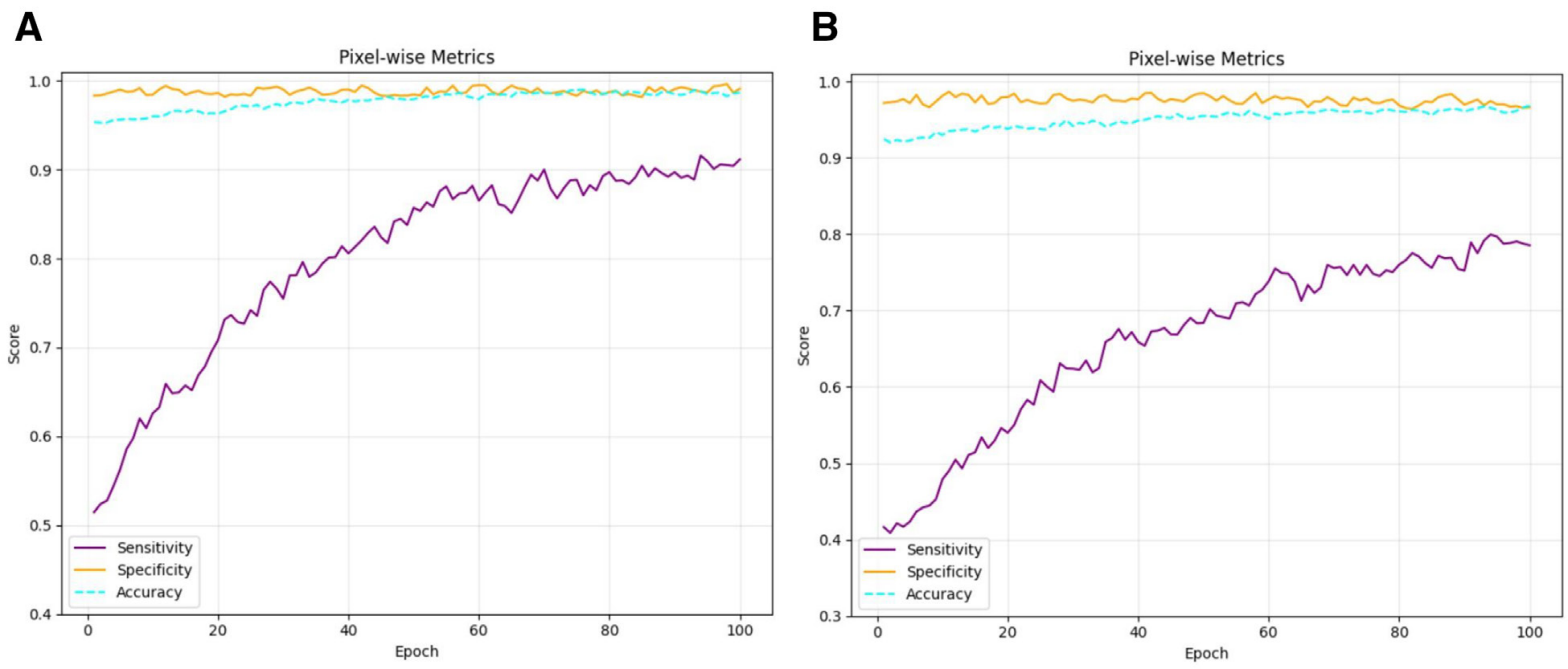
Sensitivity, specificity, and accuracy curves for both models over 100 epochs. (A) Mask R-CNN, (B) U-Net. R-CNN = regions with convolutional neural network, U-Net = U-shaped network.

The most notable improvement was observed in sensitivity. The Mask R-CNN model achieved a sensitivity of 91.16%, whereas U-Net reached only 78.54%. This 12.62% gap indicates that the proposed model is significantly more robust in identifying subtle residual root fragments that U-Net often fails to detect. The high Specificity (99.12%) further confirms that the model maintains a very low FP rate, which is critical for clinical diagnostic reliability (Fig. 6). To further validate the statistical stability of the models across different data subsets, the mean performance and 95% confidence intervals from the 5-fold cross-validation are presented in Table 3.

**Table 3:** Five-fold cross-validation performance: mean metrics and 95% CIs demonstrating model stability.

Model	Sensitivity	Specificity	Accuracy	Dice
U-Net (5-Fold Mean)	76.82% (74.5–79.1)	95.95% (95.1–96.8)	96.10% (95.4–96.8)	81.65% (79.8–83.5)
Mask R-CNN (5-Fold Mean)	89.94% (88.2–91.6)	98.85% (98.3–99.3)	98.21% (97.6–98.8)	89.80% (88.4–91.2)

CI = confidence interval, R-CNN = regions with convolutional neural network, U-Net = U-shaped network.

Statistical comparison using McNemar test confirmed that the improvement in sensitivity achieved by the Mask R-CNN over the baseline U-Net was highly significant (*P *< .001).

### 3.3. ROC curve and AUC analysis

**Figure 7.**
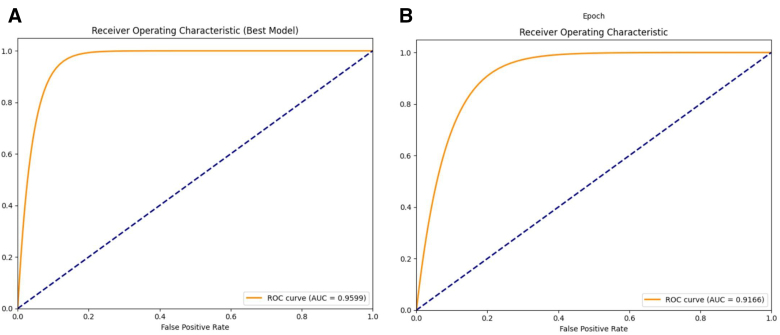
ROC curve for both models over 100 epochs. (A) Mask R-CNN, (B) U-Net. AUC = area under the curve, R-CNN = regions with convolutional neural network, ROC = receiver operating characteristic, U-Net = U-shaped network.

The diagnostic capability was further validated through ROC analysis (Fig. 7). The AUC for the enhanced Mask R-CNN was 0.9599, markedly higher than the 0.9166 achieved by U-Net. The ROC curve for Mask R-CNN shows a steeper ascent toward the top-left coordinate, representing a superior trade-off between TP and FP rates. This high AUC value underscores the effectiveness of combining Lab-space CLAHE enhancement with the multitask loss function in the Mask R-CNN framework.

## 4. Discussion

The deployment of the Mask R-CNN architecture for the detection of retained root fragments has yielded a diagnostic profile characterized by high sensitivity (91.16%) and exceptional specificity (99.12%). Interpreting these metrics requires moving beyond statistical validation to clinical translation, where the consequences of FN (missed pathology) and FP (false alarms) differ fundamentally in their impact on patient safety and surgical workflow.

The reported 12.62% improvement in sensitivity, elevating the system’s performance to 91.16%, is the most clinically significant outcome of this study. In the context of pre-implant assessment or clearances for radiotherapy, a “missed” retained root (FN) is a “never event” that can lead to catastrophic failure (such as osteomyelitis, implant non-integration, or the persistence of infectious nidi in immunocompromised patients). While accuracy is a general measure, sensitivity specifically quantifies the “safety net” provided by the artificial intelligence.^[23,24]^ By effectively reducing the FN rate, the system compensates for human limitations such as inattentional blindness and fatigue, ensuring that subtle, radiographically obscure fragments are flagged for review before irreversible surgical decisions are made.

While sensitivity ensures safety, the high specificity of 99.12% is the driver of clinical adoption and workflow efficiency. In automated diagnostic support systems, low specificity results in a high volume of FP, causing “alert fatigue” where clinicians eventually ignore or disable the software. A specificity near 99% implies that the system is “quiet” until it detects a TP, thereby maintaining the clinician’s trust. Furthermore, in a surgical context, high specificity prevents iatrogenic injury; it ensures that a surgeon is not prompted to explore healthy bone in search of a nonexistent root, preserving vital anatomy and reducing operative trauma.^[2,25]^

The superior performance of Mask R-CNN over semantic segmentation models like U-Net is rooted in its ability to decouple detection from segmentation; specifically, the RPN efficiently filters background noise to address class imbalance,^[26,27]^ while the RoIAlign mechanism preserves the pixel-level fidelity required to delineate critical features like PDL space. This architectural robustness translates into an impressive AUC of 0.9599, reflecting excellent discriminative ability where the model learns to identify invariant dentin structures rather than memorizing noise patterns. Consequently, the system effectively distinguishes true pathology from anatomical superimpositions (e.g., the mylohyoid ridge), achieving high TP rates without a commensurate increase in FP across varied patient demographics.

The study is limited by its retrospective, single-center design and restricted sample sources. This lack of data diversity risks overfitting the model to specific equipment or demographics, thereby reducing its generalizability. Secondly, the inherent nature of 2D PR fails to provide critical buccolingual depth information and suffers from geometric distortion, which compromises surgical precision. The limitations of 2D panoramic imaging for depth assessment and geometric distortion have been emphasized in cone beam CT (CBCT) comparative studies, underscoring the potential value of 3‑dimensional imaging for surgical planning.^[28]^ Finally, although the model performs well, the presence of metallic artifacts remains a challenge that could obscure or mimic root fragments. Future validation on CBCT‑derived datasets is warranted because CBCT‑based assessments provide more reliable apical/root detail and depth information for surgical planning.^[29,30]^ Furthermore, the intentional exclusion of images with severe metallic artifacts introduces a spectrum bias; the model’s performance on highly degraded clinical images remains unverified.“

## 5. Conclusion

The proposed Mask R-CNN system demonstrates improved performance for automated dental diagnostics. With a 12.62% increase in sensitivity and sustained high specificity, it provides a practical approach to identifying retained roots. These results suggest the system can effectively support surgical planning as a reliable diagnostic aid, reducing the likelihood of overlooked roots.

## Author contributions

**Conceptualization:** Jiao Zhang.

**Data curation:** Rui Li, Min Lu.

**Formal analysis:** Wenhui Li, Yaqin Chang.

**Investigation:** Wenhui Li.

**Methodology:** Jiao Zhang.

**Project administration:** Jiao Zhang.

**Resources:** Rui Li, Min Lu.

**Software:** Wenhui Li, Yaqin Chang.

**Supervision:** Jiao Zhang.

**Visualization:** Yaqin Chang.

**Writing – review & editing:** Jiao Zhang.

**Writing – original draft:** Wenhui Li.
